# Lessons Learned in a Feasibility Study With Women Caregivers of Mexican Origin

**DOI:** 10.1093/geroni/igaf122.718

**Published:** 2025-12-31

**Authors:** Hsueh-Fen Kao

**Affiliations:** The University of Texas at El Paso, El Paso, Texas, United States

## Abstract

With the rapidly aging Hispanic/Latino population and the traditional caregiving roles of women, it is critical to study caregiving stress in the largest yet understudied subgroup of women caregivers of Mexican origin. This feasibility study aimed to adapt a research protocol examining the impact of long-term caregiving stress on coronary heart disease (CHD) risk among women caregivers of Mexican origin, using the allostatic load model. A convenience sample of 20 women who provided family care (an average of 24 hours per week) for at least past six months to a dependent older relative aged 60 or older was recruited through community networks, home healthcare agencies, promotoras, the Alzheimer’s Association local chapter, and hospital outpatient services in El Paso, Texas. Key adjustments included tailoring terminology to align with participants’ preferences, managing complex data collection, and refining recruitment criteria to reflect cultural caregiving norms. COVID-related delays required further adaptations, such as proactive licensing management and alternative recruitment strategies. Inclusion and exclusion criteria were revised to better capture family caregiving dynamics, ensuring continuity despite staff turnover and rising costs due to the study’s extended timeline and inflation. By addressing these challenges, the study established a foundation for future research on women caregivers of Mexican origin. This research will focus on developing preventive interventions to reduce caregiving stress and CHD risk, ultimately supporting age-in-place for the dependent older adults in family care.

